# Glaciation Effects on the Phylogeographic Structure of *Oligoryzomys longicaudatus* (Rodentia: Sigmodontinae) in the Southern Andes

**DOI:** 10.1371/journal.pone.0032206

**Published:** 2012-03-01

**Authors:** R. Eduardo Palma, Dusan Boric-Bargetto, Fernando Torres-Pérez, Cristián E. Hernández, Terry L. Yates

**Affiliations:** 1 Centro de Estudios Avanzados en Ecología y Biodiversidad, CASEB, and Departamento de Ecología, Facultad de Ciencias Biológicas, Pontificia Universidad Católica de Chile, Santiago, Chile; 2 Departamento de Zoología, Facultad de Ciencias Naturales y Oceanográficas, Universidad de Concepción, Concepción, Chile; 3 Instituto de Biología, Pontificia Universidad Católica de Valparaíso, Valparaíso, Chile; 4 Department of Biology and Museum of Southwestern Biology, University of New Mexico, Albuquerque, New Mexico, United States of America; Barnard College, Columbia University, United States of America

## Abstract

The long-tailed pygmy rice rat *Oligoryzomys longicaudatus* (Sigmodontinae), the major reservoir of Hantavirus in Chile and Patagonian Argentina, is widely distributed in the Mediterranean, Temperate and Patagonian Forests of Chile, as well as in adjacent areas in southern Argentina. We used molecular data to evaluate the effects of the last glacial event on the phylogeographic structure of this species. We examined if historical Pleistocene events had affected genetic variation and spatial distribution of this species along its distributional range. We sampled 223 individuals representing 47 localities along the species range, and sequenced the hypervariable domain I of the mtDNA control region. Aligned sequences were analyzed using haplotype network, Bayesian population structure and demographic analyses. Analysis of population structure and the haplotype network inferred three genetic clusters along the distribution of *O. longicaudatus* that mostly agreed with the three major ecogeographic regions in Chile: Mediterranean, Temperate Forests and Patagonian Forests. Bayesian Skyline Plots showed constant population sizes through time in all three clusters followed by an increase after and during the Last Glacial Maximum (LGM; between 26,000–13,000 years ago). Neutrality tests and the “*g*” parameter also suggest that populations of *O. longicaudatus* experienced demographic expansion across the species entire range. Past climate shifts have influenced population structure and lineage variation of *O. longicaudatus*. This species remained in refugia areas during Pleistocene times in southern Temperate Forests (and adjacent areas in Patagonia). From these refugia, *O. longicaudatus* experienced demographic expansions into Patagonian Forests and central Mediterranean Chile using glacial retreats.

## Introduction

Glacial cycles that have affected the distributional range of species during the Quaternary have been the focus of multiple phylogeographic studies intending to evaluate the occurrence of single or multiple gene genealogies [1], [2], [3], [4]. For many species, Pleistocene glaciations led to continental-scale migrations and reduction in population sizes, followed by recolonization and population expansion as glaciers retreated [5], [6], [7]. Climate change induced alterations in species distribution and/or population size, promoting different scenarios of divergence due to intraspecific variation reflecting both recent and historical events [8], [9]. In addition, divergence promoted by glaciers could have left populations allopatrically distributed (i.e., in refugia), therefore we should be able to detect distinct genealogies and/or structured populations [1].

The southern Andes were repeatedly glaciated during the Late Cenozoic. In particular, the Patagonian Andes were glacier-covered prior to 4.6 mya (Late Miocene) and icefields repeatedly expanded between 2.4 and 1.2 mya [10], [11]. The greatest glaciations, however, developed during the early Pleistocene. In southern Patagonia, glaciers advanced up to 200 km east of the Andes mountains, reaching the Atlantic and the Pacific coast south of latitude 43°S [10]. The middle Pleistocene was also characterized by interglacial and glacial periods, and by strong pulses of mountain uplifts that changed glacier distributions entrenched in canyons or valleys [12], [13]. Morphostratigraphic sequences depict three major glacial advances that appear to be similar to those of the Wisconsinan glaciations that occurred in the northern hemisphere between 70,000 and 15–10,000 years ago [10].

Throughout the Quaternary, the mountain range of the Andes, the “Cordillera de los Andes” (the eastern geographic limit of Chile) experienced strong volcanic activity and glaciation events in the Temperate region [14], [15]. In contrast, the mountain range of the coast, the “Cordillera de la Costa” (the western limit of Chile that parallels the Andes) remained unglaciated and constituted a major refuge area for Temperate Forests, as well as the lowlands in the longitudinal valley between both major mountain ranges [16], [17], [18]. A strong body of evidence suggests that the patterns of distribution of present day southern forests of Chile (mostly dominated by *Nothofagus* forests, the “southern beeches”) were strongly affected by glacial cycles of the Pleistocene [19]. Approximately two thirds of the current range of southern forests were severely reduced by the Last Glacial Maximum (LGM, 13.000–26.000 years ago), particularly those that ranged south of the 43°S [20]. Glaciations therefore caused latitudinal and altitudinal shifts of forests, as well as the formation of refugia in low elevation and coastal zones [21]. North of 43°S, the Chiloé island and surrounding lakes were also glaciated, although the northwest part of the Chiloé island (Cordillera de Piuchué, 42°S) represents the southernmost emergent portion of the coastal range in Chile, and probably the nearest available refugia for the subantarctic forests and Magellanic moorland vegetation during the Pleistocene [22]. Refuge areas on the continent in southern Chile remained ice-free zones, particularly in areas of the Costal Cordillera such as Cordillera Pelada (Los Ríos region, 39°S) and Cordillera de Nahuelbuta (Araucanía region, 38°S). Intermediate zones between the latter mountains in the lowlands were also free of ice [23], [24], [25], [26], [27].

**Figure 1:**
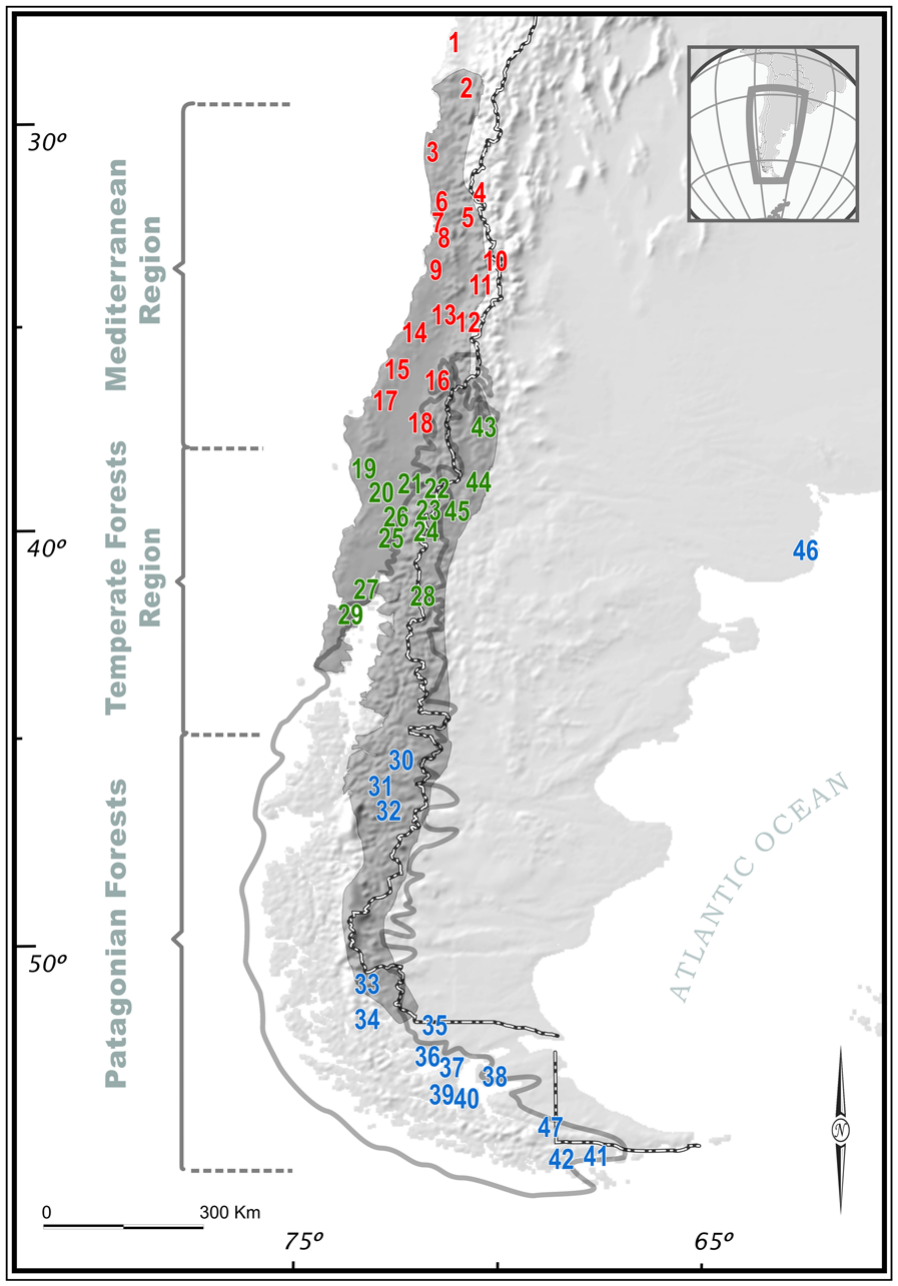
Distribution of *Oligoryzomys longicaudatus*. Geographic distribution of *Oligoryzomys longicaudatus* in Chile and adjacent zones in Argentina (gray pattern). Numbers represent sampled localities in both countries (see Table 1 for details; red numbers represent the Mediterranean ecoregion, green numbers represent the Temperate Forests ecoregion, and blue numbers represent the Patagonian Forests ecoregion). The heavy gray line shows the ice margin at the Last Glacial Maximum (LGM; after [15]).

An important component of the small mammal fauna of southern forests in Chile and adjacent areas in Argentina is the “long-tailed pygmy rice rat” (“colilargo”) *Oligoryzomys longicaudatus* (Rodentia, Sigmodontinae). Latitudinally, it ranges between 27–54°S, encompassing three of the major ecogeographic regions in the southcentral portion of Chile: the Mediterranean, the Temperate, and the Patagonian Forests ([19], [28]; Fig. 1). Altitudinally, the species ranges from sea-level to about 1000–1500 m [2], [29]. This is a small mouse distributed in the southern scrub forests, although it also is found in Patagonian and scrub forests in central Chile always associated to mesic conditions [29], [30]. Because *O. longicaudatus* is the major reservoir of the Andes strain of Hantavirus [31], [32], its wide distribution has important implications for epidemiological research. Andes Hantavirus is the predominant etiologic agent of Hantavirus Cardiopulmonary Syndrome (HCPS) in human populations of Chile and Argentina [31], [33], [34], [35], with a high mortality rate of about 35–40% (http://epi.minsal.cl). Knowledge of phylogeographic patterns of reservoir populations can provide insight into factors responsible for the occurrence and spread of the virus in natural populations [36], [37], [38], [39], [40].

*Oligoryzomys longicaudatus* occurs in areas that were glaciated during the Pleistocene, and in formerly non-glaciated zones that presumably constituted refugia (i.e., coastal and low elevations in the foothills of the southern Andes; in the Patagonian steppe of Argentina; [41]). Populations located in refugia might have served as source populations during postglacial colonization, dispersing north and southward along coastal and central valley areas in Chile. Refugia have also been postulated on the eastern side of the Andes, although through a narrow zone between the border of the ice sheet and the Patagonian steppe [10]. From this area, further postglacial colonization to the south could have occurred. It has been shown that populations in colonized areas differ genetically with respect to those of source populations [6], [7]. Source populations show higher levels of genetic richness, whereas formerly glaciated areas are expected to harbor only a subset of the source genetic variability due to range expansion and genetic drift following deglaciation [7], [42]. Alternatively, populations of colonized areas have been found to be equally or even more genetically variable than those of refuge zones because of the admixture of immigrants from different refugia [43], [44], [45].

A former phylogeographic study on *O. longicaudatus* based on cytochrome *b* mitochondrial sequences (Cyt-*b*; [2]) supported previous results based on morphology, chromosomes and allozymes, suggesting genetic uniformity of the species along its range [2], [46], [47]. The high vagility of this species, particularly in mesic regions of southern Chile [48], together with recent postglacial colonization, have been suggested to have contributed to the genetic homogeneity among populations [2]. However, recent epidemiological works related to Hantavirus, which included additional specimens and Cyt-*b* sequences of *O. longicaudatus* (to that of [2]), showed spatial subdivision in the latitudinal range [38], [40]. Indeed, three ecogeographic subunits were recovered from north to south of the geographic range of the species: Mediterranean, Temperate Forests and Patagonian groups according to the homonymous ecoregions.

This study evaluates the genetic consequences of Pleistocene glaciations on the phylogeographic structure of the “long-tailed pygmy rice rat” *Oligoryzomys longicaudatus*. Since the species is distributed in glaciated and non-glaciated areas, we analyzed the effects that glaciation events had on *O. longicaudatus* populations along its range in south-central Chile and adjacent Argentina. Specifically, we tested if populations of the long-tailed mouse experienced demographic expansion from refugia in southern Chile, and discuss the biogeographic scenario that yielded its current phylogeographic structure. To achieve these goals, we sequenced the hypervariable domain I of the mitochondrial DNA (mtDNA) *control region*, and aligned sequences were analyzed using Bayesian approaches.

## Results

A total of 331 bp of the mitochondrial DNA control region (CR), *hypervariable region I* (HVI), were sequenced from 223 specimens representing 47 localities in Chile and Argentina (Fig. 1, Table S1). We recovered 89 polymorphic sites and 117 haplotypes throughout the analysis, of which 50 haplotypes were from the Mediterranean ecoregion, 32 from the Temperate Forests, 28 from Patagonia, and seven were shared haplotypes (Table S2).

**Figure 2:**
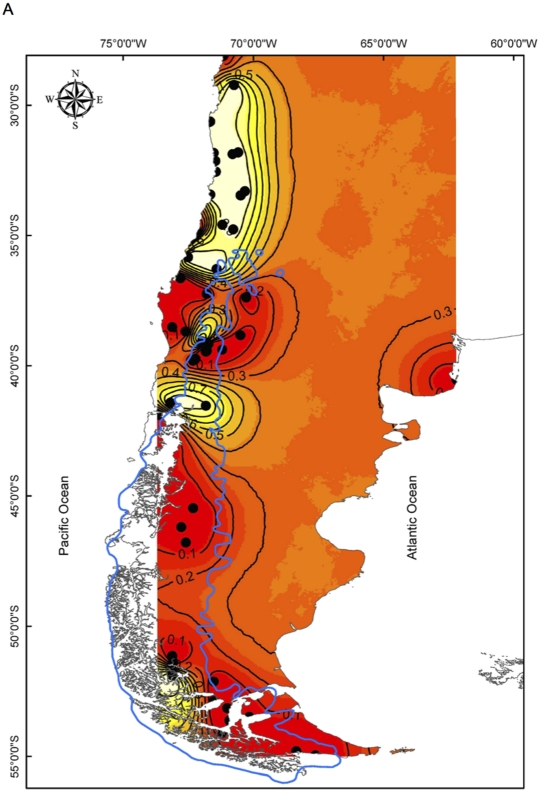
GENELAND analyses with posterior probability isoclines denoting the extent of genetic landscapes. The Mediterranean (2a), the Temperate Forests (2b) and Patagonia (2c) ecoregions are recovered in the figure. To facilitate interpretation, GENELAND output has been cropped, re-scaled and superimposed over the map of Chile and Argentina where *O. longicaudatus* ranges. Black dots represent localities analyzed in this study and the limit of the LGM is also shown. Regions with the greatest probability of inclusion are indicated by white, whereas diminishing probabilities of inclusion are proportional to the degree of coloring.

The population structure for *O. longicaudatus* was evaluated using the GENELAND v. 1.0.7 program [49], [50], [51]. The analyses inferred three genetic clusters in the study area that mostly agreed with the three major ecogeographic regions of Chile, although each of them also included some localities of other ecoregions. In fact, the first cluster mostly grouped sequences representing localities of Mediterranean Chile between 30–36°S, but it also included samples of three localities from the Temperate Forests (localities 21, 27 and 28 in map Fig. 1; see black dots in Fig. 2a), and a sample from Patagonia (locality 34 in map Fig. 1; see black dot in Fig. 2a). The second cluster joined sequences representing localities of the Temperate Forests of southern Chile, three localities from Argentinean Temperate Forests (43–45 in map Fig. 1) between 38–45°, and two localities from Patagonia (localities 30 and 47 in map Fig. 1; see black dots in Fig. 2b). Within the latter cluster some localities are within the LGM area (Fig. 1), whereas others fell at both sides of the LGM area in the Chilean and the Argentinean sides (Fig. 2b). The third cluster of sequences included Patagonian localities from 45°S southward, within the limits of LGM (Fig. 2c). In addition, the Patagonian cluster included the Argentinean locality of San Blas (Buenos Aires province; Fig. 2c).

**Figure 3:**
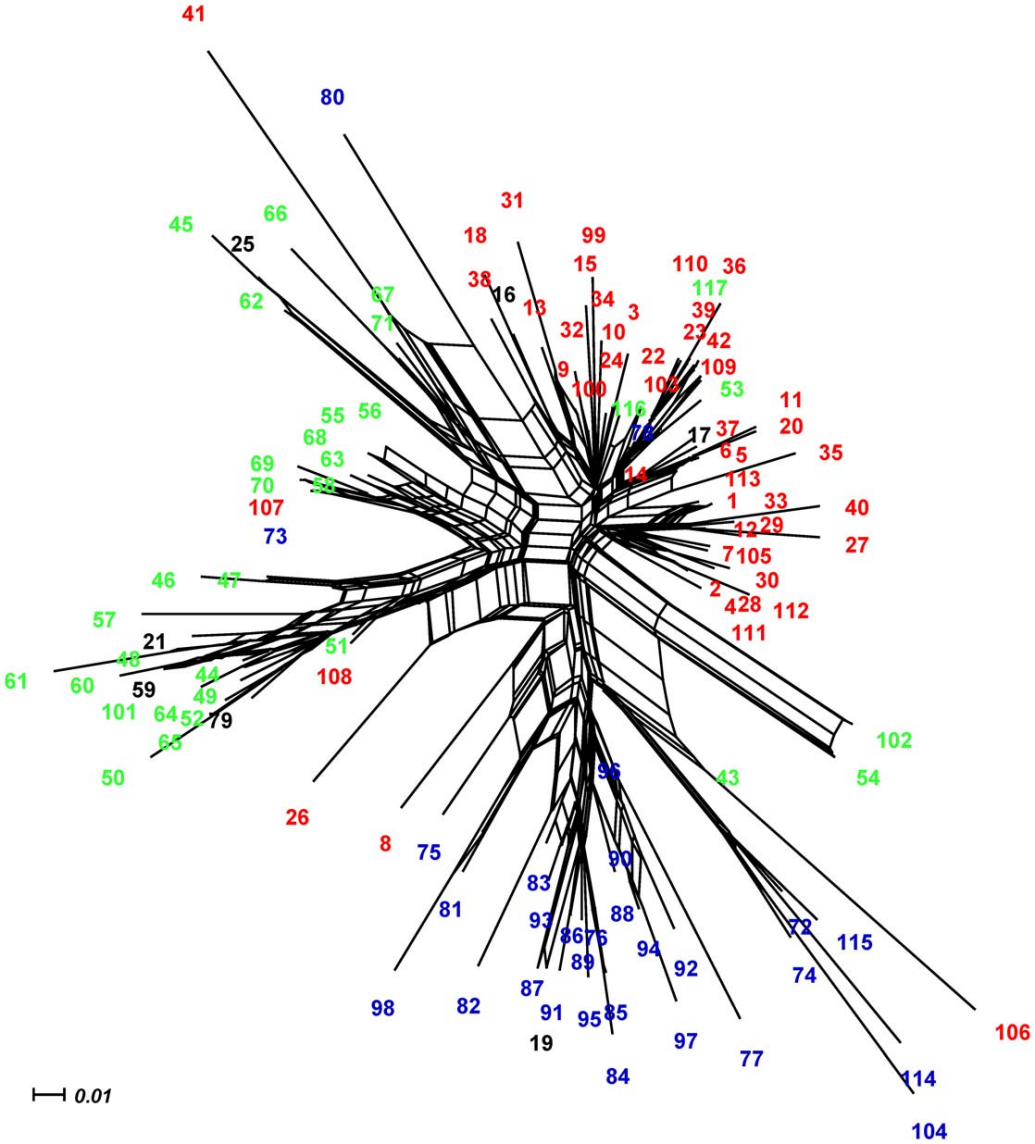
Neighbornet of HVI haplotypes. Labels for haplotypes represent the following groups: red = Mediterranean ecoregion, green = Temperate Forest ecoregion, blue = Patagonian ecoregion.

Regarding segregating sites per cluster, we found 51 in Cluster 1 Mediterranean, 46 in Cluster 2 Temperate Forests, and 47 in Cluster 3 Patagonia. The three groups showed similar haplotype diversity although Patagonia was slightly higher, whereas overall nucleotide diversity was low especially for Mediterranean Chile. The haplotype diversity (Hd) was 0.967 for Cluster 1 Mediterranean, 0.968 for Cluster 2 Temperate Forests, and 0.991 for Cluster 3 Patagonia (Table 1). The nucleotide diversity (pi) was 0.014, 0.022, and 0.023, respectively. The Neighbor-Net network shows three major lineages diverging from an unresolved polytomy (Fig. 3). These lineages represent the Mediterranean (red), the Temperate Forests (green) and the Patagonian Forests (blue) ecoregions. Shared haplotypes between different ecoregions were depicted in black (Fig. 3). The Mediterranean and the Temperate Forests ecoregions shared five haplotypes (haplotypes 17, 21, 25, 59 and 79; Fig. 3, Table S2), whereas the Mediterranean and Patagonia ecoregions shared two haplotypes (haplotypes 16 and 19; Fig. 3, Table S2). We did not find shared haplotypes between the Temperate and Patagonian Forests.

**Table 1:** Descriptive statistics of genetic variation and Fu-*Fs*, Tajima's D and Ramos-Onsis & Rozas' R_2_ neutrality tests of *O. longicaudatus* sequences in Chile and Argentina.

Cluster	N	S	Hd ± SD	PI ± SD	Fu-*Fs* (P-value)	Tajima's *D* (P-value)	R_2_ (P-value)	Θ_G_ [95% confidence interval]	G [95% confidence interval]
Mediterranean	111	51	0.967±0.006	0.014±0.001	−45.901 (0.000)	−1.725 (0.015)	0.046 (0.018)	0.2454 [0.1323, 0.4481]	252.8 [132.4, 523.2]
Temp. Forests	78	46	0.968±0.008	0.022±0.001	−19.139 (0.001)	−0.704 (0.273)	0.085 (0.375)	0.1388 [0.0836, 0.2397]	118.2 [34.88, 248.0]
Patagonia	34	47	0.991±0.010	0.023±0.002	−19.567 (0.000)	−1.247 (0.084)	0.077 (0.093)	0.6527 [0.2645, 2.5055]	246.4 [141.9, 483.2]

*N*: Number of individuals; S: Number of segregating sites; Hd: Haplotype diversity; pi: Nucleotide diversity; Θ_G_ = a compound parameter representing the effective population size and substitution rate estimated under the model of growing population; g: Growth parameter (*t* = 1/μ); SD: Standard deviation.

**Figure 4:**
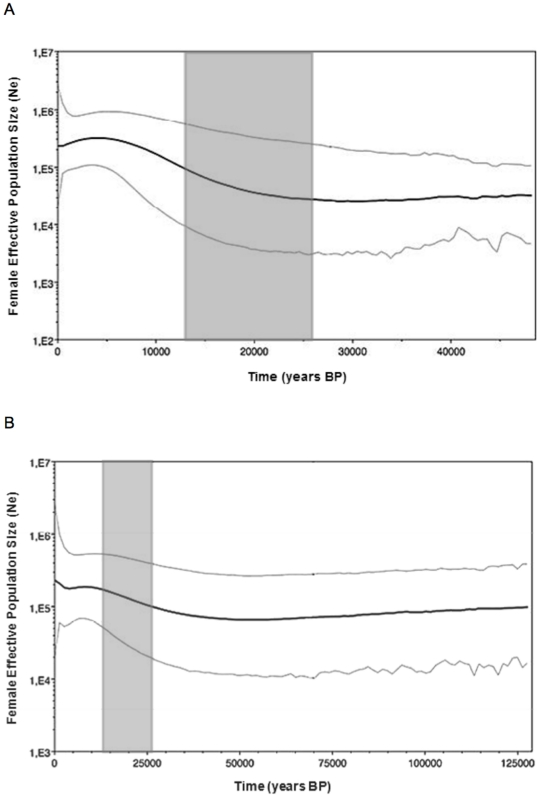
Bayesian Skyline Plots (BSP) for each GENELAND cluster. The historical demographic trends of mitochondrial lineages in *Oligoryzomys longicaudatus* are represented. The x axis is the time in 10^3^ years before present; the y axis is the estimated female effective population size. Fig. 4a shows the BSP for Cluster 1 Mediterranean ecoregion, Fig. 4b for Cluster 2 Temperate Forests, and Fig. 4c for Cluster 3 Patagonia ecoregions. The grey areas shows the Last Glacial Maximum (LGM) between 26,000 and 13,000 years [20].

We also performed historical demographic reconstructions through the Bayesian Skyline Plots (BSP). After a period of constant population size, analyses showed an increase in the effective population size starting about 12,000 years ago for the Mediterranean cluster (Fig. 4a), about 26,000 years ago for the Temperate Forests cluster (Fig. 4b), and about 25,000 years ago for Patagonia (Fig. 4c). Finally, Fu's Fu neutrality test statistics (Table 1) were negative and significantly different from zero for all clusters: Mediterranean (−45.901, *P* = 0.000), Temperate Forests (−19.139, *P* = 0.001), and Patagonia (−18.321, *P* = 0.000), indicating that the null hypothesis of population equilibrium is rejected in favor of a population expansion. Maximum likelihood estimations of the ‘*g*’ parameter were positive and significant (at the 95% of confidence interval) for each of the clusters along the *O. longicaudatus*'s range (Table 1): Mediterranean (252.8 [132.4–523.2]), Temperate Forests (118.2 [34.88–248.0]), and Patagonia (246.4 [141.9–483.2]). These results also suggest that the hypothesis of a stable demographic history is rejected in favor of a recent expansion (Table 1).

## Discussion

### Haplotype and nucleotide diversity

*Oligoryzomys longicaudatus* has high haplotype diversity (all clusters are at or close the maximum value of 1.00), but low nucleotide diversity for each of the clusters, suggesting recent differentiation with rapid population growth [52]. This is particularly true for Patagonia where we obtained the highest haplotype diversity, and in the Mediterranean ecoregion where we recovered the lowest nucleotide diversity. A similar pattern of nucleotide and haplotype diversity was found using partial Cyt-*b* mitochondrial sequences in a previous study [2], where nearly every locality across the “long-tailed mouse” range contained a unique haplotype. This pattern of genetic variability suggests population growth following a period of reduced effective population size [1], [52], [53]. We observed low genetic diversity in most of the surveyed populations, but analyses of within-ecoregion (or cluster) diversity did not reveal whether this is due to recent origin (founder effect) or due to large fluctuations in abundance (bottleneck). The consequences of these two processes in terms of extant genetic diversity can be nearly identical and thus hard to distinguish [52]. Low genetic divergence among populations, as reflected by low values of nucleotide diversity among haplotypes, suggest that postglacial colonization into recently available central ranges occurred rapidly [52]. Indeed, our BSP analyses for the three clusters supported recent expansion for *O. longicaudatus* between 13,000 (for the Mediterranean ecoregion, cluster 1) and 25,000–26,000 years ago (for the Temperate and Patagonia ecoregions, clusters 2 and 3), which is in the range of the LGM event [54]. Evidence of demographic expansion for each cluster was also supported by results of the Fu Fs neutrality test and the ‘*g*’ parameter. According to our GENELAND results that recovered localities in glaciated and non-glaciated areas of southern Chile, we hypothesize that populations of the long-tailed mouse may have dispersed from these areas that might have constituted refugia in the Temperate Forests and Patagonia.

### Biogeographic scenario

At least three events of glacial cycles have been reported for southern Patagonian Forests that affected the biota of the area [15] and the primary habitat for *O. longicaudatus* [29]. Since a great part of Patagonia was glaciated, palynological evidence suggests the occurrence of habitat expansions to the north through the central valley and the Coastal Cordillera. While glaciations advanced to the north throughout the Cordillera de los Andes, refugia may have remained in the central valley between the Andes and the Coastal Cordillera. Pollen records suggest the southern coastal range of lowland Chile and the Chiloé island, as well as the southern central depression, as the most likely locations of refugia between 40–42°S [18], [21], [24], [55]. The latter areas served as refugia to diverse taxa such as the long-lived conifer *Fitzroya cuppresoides* [56], the Andean Asteraceae *Hypochaeris palustris* [57], the Nothofagaceae tree *Nothofagus pumilio* [58], some lizards of the genus *Liolaemus* [4], the fresh water shrimp *Samastacus spinifrons* (Castro *et al.*, unpublished), and the sigmodontine mice *Abrothrix olivaceus* [59], [60], [61] and *A. longipilis* [62]. The latter two species are commonly found coexisting with *O. longicaudatus* along its distribution in Chile and adjacent Argentina. Glacial cycles strongly affected Temperate Forests, the primary habitat of *Oligoryzomys*, which may have disturbed local populations of this species. Our results support the hypothesis that postglacial colonization of *O. longicaudatus* probably occurred from multiple refugia in southern Chile between 26,000 to about 13,000 ya, which is in agreement with palynological data [19]. These areas were mainly located in the coast and in the central depression zones that were not reached by glaciers.

GENELAND and haplotype network analyses recovered three major clusters along the distribution of *Oligoryzomys longicaudatus* that closely correspond to the Mediterranean, the Temperate Forests and Patagonian steppes and forests. Since our results suggest that Temperate Forests samples constitutes the ancestral haplotypes because they are scattered through the network, we hypothesize that expansion of populations might have occurred from southern refugia, such as the localities assigned to the Mediterranean cluster within and outside the limits of the LGM (Fig. 2a). The second major clustering grouped samples from the Temperate Forests between 36–40°S, and includes localities inside and outside the LGM (Fig. 2b). Localities outside the LGM are from the east and west of the glacial maxima, in the Argentinean and Chilean side, respectively. Localities within the limits of LGM suggest that these populations were colonized after the glacial retreat or remained in refugia during glaciation (i.e., locality 47, Ushuaia in Fig. 1). The recolonization of glaciated areas may have occurred from refugia located in the central depression and the coastal areas of Chile, in the south of the country (i.e., locality 27, Puerto Montt in Fig. 1), and from localities outside the limits of glaciers in Argentina. The last major clustering of samples grouped the Patagonian localities south of 45°S, including those from the Aysén and Magallanes regions in Chile, and the Argentinean locality of San Blas, in the province of Buenos Aires. All localities recovered in this cluster were placed within the limits of the LGM, and due to higher molecular variation (Table 1), the current structure of population probably is not the result of a simple colonization event. However, recolonization may also have occurred from possible refugia inside limits of the LGM (i.e., Reserva Alacalufe, Ushuaia).

We hypothesize that populations of *O. longicaudatus* remained in refugia mainly on the coast and the central depression of Chile, about latitudes 39–40°S, until glaciers retreated, followed by rapid dispersal to the north and south of these latitudes. Several refugia have been proposed in southern and Patagonian Chile, east and west to the Andes [41], [56], [59], [63], [64], [65], [66], [67], [68]. These results contrast with those of a recent study that proposed dispersal for several Patagonian sigmodontine taxa to higher latitudes from “single restricted sources” [69]. Instead, our data suggest the occurrence of multiple refugia located primarily in the Temperate Forests and Patagonia. The high vagility and large home range of *O. longicaudatus*, particularly in southcentral and Patagonian Chile [48], would have facilitated rapid dispersal within the last 25,000 years. GENELAND analyses show that some individuals cluster with those from different ecoregions. For example, Temperate Forest samples from Lago Colico (21), Puerto Montt (27) and Paso El León (28) grouped together in the Mediterranean cluster 1 with Reserva Nacional Alacalufe (34) from Patagonia (Fig. 2a), whereas the Mediterranean samples from Llanos de Challe (1) and Duao (14) grouped together with Patagonian samples from Río Simpson (30) and Tierra del Fuego (47) in the Temperate Forest cluster 2 (Fig. 2b). These results may reflect retention of ancestral polymorphisms [70], which would have persisted in southern and eastern refugia. In fact, the area of Puerto Montt, Reserva Nacional Alacalufe, and Tierra del Fuego have been proposed as refugia [21], [54], [61]. Samples from Río Simpson deserve particular attention because some of their sequences were assigned to the Temperate Forests cluster, and others to the Patagonia. This might be explained from a secondary contact after glacial retreat from refugia or unglaciated area. Indeed, in the Patagonia cluster is where we found higher haplotypic diversity and private haplotypes suggesting refugia in Patagonia.

The same scenario of post-colonization might have occurred from coastal refugia to Patagonian localities. However, the latter sites showed higher nucleotide variation against predictions for post-glaciated areas [6], [71], [72]. We would have expected a lower nucleotide diversity in Patagonian post-colonized sites compared to refugia. Comparative phylogeographic analyses in the northern hemisphere have revealed the role of refugia in harboring genetic diversity of mammal populations during glaciation events [71], [73], [74], [75], [76], [77]. Alternatively, genetic variation could have increased due to admixture of individuals from different source populations of Chile and Argentina during the last 15,000 years [43]. The high vagility and large home range reported for *O. longicaudatus*, particularly in southcentral and Patagonian Chile [48], would have facilitated the admixture. This scenario is supported by results of the Network analysis, which reveals that Patagonia has the highest amount of singletons separated by several mutational steps (Fig. 3).

In temperate areas of North America, dispersal of mammals to lower latitudes during glacial periods followed by demographic expansion during warm interglacials is well documented [78], [79]. There are no similar studies for temperate South America, but here we show dispersal of *O. longicaudatus* to lower latitudes in Chile (i.e., Mediterranean Chile) due to global cooling in the Pleistocene. For example, one hypothesis to explain the current relictual distribution of Temperate Forests in the coast of central Chile (i.e., Parque Nacional Fray Jorge, about 30°S), which are currently separated by more than 1,000 km from southern forests, is that they are remnants of southern vegetation that expanded during glaciations [28]. Thus, post-colonization might have allowed the forest and associated fauna to reach the north boundary of its current range multiple times following glacial pulses of the Pleistocene. This northern expansion hypothesis is supported by our results showing the occurrence of shared haplotypes between the Mediterranean and Temperate Forests ecoregions, and between the Mediterranean and Patagonian ecoregions, whereas between the Temperate and Patagonian Forests we did not find shared haplotypes. Thus, these sequences would represent population relicts as a result of population expansion and contraction due to glacial cycles.

Regarding the biogeographic history of *O. longicaudatus* west of the Andes, former studies have suggested a colonization from the south (Temperate Forests) to Patagonia and to the central-north of Chile [2], [46]. Our results are not congruent with a recent hypothesis suggesting the passage of *O. longicaudatus* through the Andes in a west-east direction [80]. *O. longicaudatus* is part of an Andean-Chacoean clade [81] that includes *O. andinus* from the highlands Andes of Peru and Bolivia, *O. fornesi* from Bolivia, northern Argentina, southern Brazil, and the Paraguayan Chaco [82], [83], and an unnamed taxon called “species B” from the Peruvian Andes (Arequipa). We sustain that this Andean-Chacoean group diversified about 2.2 mya from an Andean-Patagonian ancestral distribution [81], leaving *O. andinus* and *O*. spB in the highlands of the central Andes, *O. fornesi* in the foothills of the Andes and Chaco region, and *O. longicaudatus* in the southern lowland that ranges from the Andes mountains of Argentina and Chile, southward to Patagonia [81]. Gonzalez-Ittig *et al.* [80] based their hypothesis on the high nucleotide diversity contained within *O. longicaudatus* on populations west of the Andes, but they only included 4 localities (11 individuals) from the Chilean side. Their hypothesis is also based on the biogeography of another sigmodontine rodent, *Abrothrix olivaceus*, whose populations may have crossed the lower elevations of the Andes through the *Nothofagus* forests located in southern Chile and Argentina (about 40°S) in an west-east direction. The biogeographic history of *O. longicaudatus*, however, is different to that of *A. olivaceus*. While *A. olivaceus* ranges from southern Peru southward to Patagonia, *Oligoryzomys longicaudatus* ranges from the southern limit of the Atacama Desert (i.e. Llanos de Challe, locality 1) southward to Patagonia. Thus, we believe that the most parsimonious hypothesis for the entrance of *O. longicaudatus* into the Chilean Andes is from the east (Argentina), entering through the lower elevations of the Andes at about 40°S. Following predictions from the coalescent theory [84], older haplotypes would be expected to be broadly geographically distributed [85]. In our Neighbour-Net results haplotypes from Temperate Forests apparently were more broadly distributed through the tree (Fig. 3), suggesting that southern populations of *O. longicaudatus* are ancestral to central Chile and Patagonian populations, and that the entrance of the species into the Chilean side could have been from the south.

### Taxonomic implications

The three genetic clusters recovered within *O. longicaudatus* through GENELAND and Neighbor-net analyses are in agreement with the major ecogeographic regions recognized in Chile, the Mediterranean, the Temperate Forests and the Patagonian Forests. These three groupings concur with the three subspecies originally proposed by Osgood [30] for *O. longicaudatus* in each of these ecoregions, respectively: *O. l. longicaudatus*, *O. l. philippii* and *O. l. magellanicus*. An earlier study discarded the occurrence of subspecies within *O. longicaudatus* [46], leaving *longicaudatus* and *philippii* as a synonym of the former and recognizing the species *O. longicaudatus* (Mediterranean and Temperate Forests) and *O. magellanicus* (Patagonia). However, we suggest that the latter proposition should be revised. *Oligoryzomys longicaudatus* has a broad distribution in Chile, south of the Atacama Desert (about 27°S) downwards to the Patagonia including Tierra del Fuego (Chile and Argentina), and the Navarino Island (about 55°S; [86]. Besides the structuring found in *O. longicaudatus*, some mixing of populations is also true as shown in GENELAND analysis, that could be due to ecological (i.e., high vagility reported for this species), and historical effects (dispersal from potential refuge areas in southern Chile and Argentina) [87]. The karyotipic uniformity along the geographic range of *O. longicaudatus* [2], [86] backs the flow of individuals along the geographic range. Our results confirm the three geographic subspecies proposed by Osgood [30] and Mann [29] based on morphology and now supported by molecular data. As we stated above, each of the subspecies is restricted to a major ecoregion: *O. l. longicaudatus* to the Mediterranean ecoregion between Atacama and Biobio (27–38°S), *O. l. philippii* to the Temperate Forests (38–45°S), and *O. l. magellanicus* to the Patagonian Forests (45–55°S). As of *O. l. pampanus* from Bahía San Blas, Buenos Aires, Argentina, this taxon was recovered within the Patagonian cluster, but further analyses will be necessary to uncover the taxonomic status of this form.

### Conclusions

Our analyses suggest that populations of *Oligoryzomys longicaudatus* remained in multiple refugia within the Temperate Forests and Patagonia ecoregions of Chile and Argentina, and inside and outside of LGM icesheet. Subsequent dispersal to the north and south of previously proposed refugia and non-glaciated areas may have occurred during and after the LGM. Our results supported a population expansion scenario in the time period of glacial retreats, which may have been facilitated by the dispersal capabilities of the species. In addition, the low nucleotide diversity found among *Oligoryzomys* haplotypes suggests a recent history of expansion with high admixture between populations. A hypothesis of expansion from multiple refugia in the southern range of the species, and northern expansion due to global cooling during Pleistocene glaciation, with further retraction towards the south, was supported.

## Materials and Methods

### Specimens analyzed, study sites and ethics statement

Voucher specimens sequenced in this study were deposited in the Colección de Flora y Fauna Profesor Patricio Sánchez Reyes (SSUC), Departamento de Ecología, Pontificia Universidad Católica de Chile, Santiago, Chile; Colección de Mamíferos del Centro Nacional Patagónico (CENPAT), Puerto Madryn, Argentina (field numbers UP and LB); the Museum of Southwestern Biology (MSB), Department of Biology, University of New Mexico; and the Instituto de la Patagonia (CZIP), Universidad de Magallanes, Chile.

We analyzed 223 samples from 47 localities throughout the range of *O. longicaudatus*, representing 42 localities in Chile and five in Argentina; Fig. 1; Table S1. Although a previous work on the phylogeography of *O. longicaudatus* is available [2], this time we considerably increased the sample size per locality, the number of localities, and employed a more variable molecular marker that is the HVI mtDNA control region. Details of localities along the geographical range of *O. longicaudatus* in Chile and Argentina are given in Table S1. Of the 47 localities, 18 were located in the Mediterranean region, 14 in the Temperate Forests and 15 in Patagonian Forests. Specimens analyzed are available upon request. All sequences have been submitted to the GenBank under accession numbers [EU592967–EU593150].

We followed the guidelines of the American Society of Mammalogists during the collection and handling of the animals used in this work [88], as well as the biosafety procedures for handling specimens with Hantavirus [89]. This research was undertaken with approval from the Animal Welfare Assurance from the National Institutes of Health, Public Health Service (USA), allowing to work with vertebrate animals (Assurance # A5848-01).

### Laboratory procedures

DNA was extracted from frozen liver samples using QIAamp Mini Kit (QIAGEN™ Inc., Valencia, CA, USA). The HVI mtDNA control region was amplified via polymerase chain reaction (PCR) using primers DLO-L (CGGAGGCCAACCAGTAGA-3)′ and DLO-H (TAAGGCCAGGACCAAACC-3′). The PCR program consisted of an initial denaturation time of 3 min at 94°C followed by 25 cycles of denaturation for 30 s at 94°C, annealing for 30 s at 57°C, and extension for 1 min 30 s at 72°C. Double-stranded PCR products were purified with QIAquick PCR Purification Kit (Qiagen). Cycle sequencing was performed using primers DLO-L and DLO-L2 (CACTTGGGGGTGTCTAACC-3′) labeled with the Big Dye Terminator kit of Perkin Elmer (Norwalk, Connecticut, USA). Sequencing reactions were analyzed using an Applied Biosystems Prism 310 (Foster City, California, USA) automated sequencer, and sequences were aligned using the ClustalW program [90], and by eye.

### Data analyses

We used the DnaSP v 5.10.01 software [91] to describe the genetic diversity in all groups and the complete data set. We calculated the number of haplotypes (Nh), the haplotype diversity (Hd), the nucleotide diversity pi (the average number of pairwise nucleotide differences per site [92], and the segregating sites (S; Table 1). To evaluate the presence of population structure for *O. longicaudatus*, we used the program GENELAND v. 1.0.7 [49], [50], [51] in the R-Package [93], which implements a population statistical model with Bayesian inference in a set of georeferenced individuals with sequences data (http://www2.imm.dtu.dk/~gigu/Geneland/#). This model's objective is to infer and locate the genetic discontinuities between populations of geo-referenced genotypes, considering the uncertain localization of the sampled individuals. The number of clusters was determined by running MCMC (Markov chain Monte Carlo) iterations five times, allowing *K* (i.e., the most probable number of populations) to vary, with the following parameters: 5×10^6^ MCMC iterations, maximum rate of the Poisson process fixed to 100 (this is the default value of the software), uncertainty attached to the spatial coordinates fixed at 5 km to account for the possibility that inaccurate coordinates were provided, and considering that the maximum home range reported for *O. longicaudatus* is 4.5 km [48]. The minimum K = 1, maximum K = 10 (values that allow us to explore a wide potential number of populations, and considering the maximum spatial subdivision in the latitudinal range described by [38]. The maximum number of nuclei in the Poisson-Voronoi tessellation was fixed to 300 (3× maximum rate was suggested by [50]. After inferring the number of populations in the data set from these five runs, the MCMC was run 30 times with *K* fixed to the inferred number of clusters, with the other parameters the same as above. The mean logarithm of the posterior probability was calculated for each of the 30 runs and the posterior probability of population membership for each pixel of the spatial domain was then computed for the three runs with the highest values.

To establish the relationships between haplotypes, we constructed a haplotype network using the Neighbor-Net [94] distances transformation and equal angle splits transformation [95]. Splits computed from the data are represented as parallel edges rather than single branches, allowing visualization of ambiguous and conflicting signals in the data set providing an implicit representation of evolutionary history [96]. To estimate the demographic history of lineages within *O. longicaudatus* we used a Bayesian coalescent-based framework. Coalescence explains how population genetic process through time have shaped an allelic or haplotypic genealogy [97]. Based on coalescence, it is possible to infer relevant population parameters (i.e., population sizes) from genetic sequence data.

We used the coalescent-based Bayesian Skyline Plot approach (BSP; [98]) as implemented in the software BEAST version 1.5.4 [99] to estimate the demographic history. The BSP fits different demographic scenarios, minimizing the overparameterization of the demographic function. However, unlike other skyline methods, the BSP is coupled with an MCMC sampling procedure, therefore estimating the demographic parameters directly from the sequences and not from a generated genealogy. The BSP estimates a posterior distribution of trees and population parameters under the best fit substitution model. These distributions are then used to generate credibility intervals [highest posterior densities (HPD)] that represent both, phylogenetic and coalescent uncertainty [98]. BSP was performed for each of the clusters, recovered with GENELAND. The running conditions include 10 million iterations. Burn-in and convergence of the chains were determined with a 0.1 cutoff value. Starting operators were based on default settings and auto-optimized during searches of parameter space using MCMC sampling procedures. Parameter estimates were based on posterior probability distributions constructed by sampling the stationary distribution for 10,000,000 generations, sampling every 1000 steps. The measures of effective sample sizes for each parameter were found to exceed 100 individuals, which is the minimum recommended effective sample size [100]. To determine the best model of sequence evolution for each cluster we used jModeltest [101]. The selected models of molecular evolution for each cluster were: a general model of reversible time with invariable sites, and gamma distribution with four discrete categories (GTR+I+Ã) for cluster 1, a general model of reversible time (GTR) for cluster 2, and the Hasegawa-Kishino-Yano with invariable sites, and gamma distribution for cluster 3 (HKY+I+Ã). Bayes factors [102] were used to choose between strict, lognormal or exponential clock models, choosing the exponential (cluster 1 log *BF* = 3.073, cluster 2 log *BF* = 6.82 and cluster 3 log *BF* = 6.03). The Bayes factor compares two posterior probability distributions, by asking which is larger after having adjusted differences between the models (M_i_ vs M_j_) in terms of the numbers of parameters that they use. Given marginal likelihoods for two different models, the log-Bayes factor is defined as log BF = −2 log [p (D|M_i_)/p (D|M_j_]. Intuitively the Bayes factor test compares which of the models accounts for a higher proportion of the total probability of the data [p(D)]. We followed previously reported criteria to determine the support for the favored model [95]. Log-Bayes factors of zero indicate equivalence of two models, and less than 0 provide evidence for model *j*. The marginal likelihood was estimated using the method previously described [94] with modifications [103]. The Bayes factors test was performed using the software Tracer v1.5 [104]. Subsequent Bayesian analyses used the model supported by the Bayes factor test (exponential molecular clock). A substitution rate of 40% per site per million of years was chosen for the control region, estimated for *Mus musculus* [105] and used for other rodents [106]. Demographic plots for each analysis were visualized using Tracer v1.5 [104]. We assessed convergence and mixing of MCMC chains by running two independent analyses starting from different, random trees, and based on the effective sample size parameter (ESS) using Tracer v1.5 [104].

The program LAMARC 2.1.3 [107] was used for each GENELAND cluster to make simultaneous estimates of Θ = 2*N_e_*μ (measure of effective population size and per-site neutral mutation rate) jointly with the population growth rate ‘*g*’, assuming an exponential model of growth, and using the Bayesian MCMC sampling. The ‘*g*’ parameter reflects the exponential growth model; positive values of ‘*g*’ indicate (population) growth, and negative values indicate population shrinkage. We set the program to compute the Watterson estimate of Θ, and allowed the population to change in size, with an initial value for ‘*g*’ set to 0.1, a random starting tree, and the transition/transversion ratio obtained from jModelTest [101]. We ran 10 short MCMC simulations of 10,000 generations each with a sampling increment of 20 generations, and 4 long MCMC simulations of 100,000 generations each and a sampling increment of 20 generations to explore the solution space. Analyses were repeated four times with heating [108], and with different random number seeds to assess consistency. We also assessed demographic history of the resulting groups from GENELAND analyses by performing Fu's Fs neutrality test statistics [109], Tajimas's D test [110], and R_2_ [111] and testing the significance of the statistics from 10,000 simulated samples (Table 1), using DnaSP 5.10.01 [91].

## Supporting Information

Table S1 **List of geographic localities analyzed in this study.** Refer to numbers depicted in the map for localities (Fig. 1). N = sample size for that locality.
(DOCX)

Table S2 **List of haplotypes recovered through the mitochondrial control region (hypervariable I domain) for** ***Oligoryzomys longicaudatus*** **.** In parenthesis the number of haplotypes. The localities (see Table 1 for geographic details) are associated to specimens trapped in that site. The NK is a collection number used for the Division of Biological Materials of the Museum of Southwestern Biology, University of New Mexico, USA, and the Departamento de Ecología, Pontificia Universidad Católica de Chile, Chile. Other acronyms used are UP (field catalog of Dr. Ulyses Pardiñas), JCT (Juan Carlos Torres-Mura).
(DOC)

## References

[1] Avise JC (2000). Phylogeography: The History and Formation of Species.

[2] Palma RE, Rivera-Milla E, Salazar-Bravo J, Torres-Pérez F, Pardinas UFJ (2005). Phylogeography of *Oligoryzomys longicaudatus* (Rodentia: Sigmodontinae) in temperate South America.. J Mammal.

[3] Himes CMT, Gallardo MH, Kenagy GJ (2008). Historical biogeography and post-glacial recolonization of South American temperate rain forest by the relictual marsupial *Dromiciops gliroides*.. J Biogeogr.

[4] Victoriano PF, Ortiz JC, Benavides E, Adams BJ, Sites JW (2008). Comparative phylogeography of codistributed species of Chilean *Liolaemus* (Squamata: Tropiduridae) from the central-southern Andean range.. Mol Ecol.

[5] Hewitt GM (2004). Genetic consequences of climatic oscillations in the Quaternary.. Philos Trans R Soc Lond, Ser B: Biol Sci.

[6] Hewitt G (2000). The genetic legacy of the Quaternary ice ages.. Nature.

[7] Hewitt GM (1996). Some genetic consequences of ice ages, and their role in divergence and speciation.. Biol J Linn Soc.

[8] Barton NH, Wilson I (1995). Genealogies and Geography.. Philos Trans R Soc Lond, Ser B: Biol Sci.

[9] Templeton AR, Routman E, Phillips CA (1995). Separating population structure from population history: A cladistic analysis of the geographical distribution of mitochondrial DNA haplotypes in the Tiger Salamander, *Ambystoma tigrinum*.. Genetics.

[10] Rabassa J, Clapperton CM (1990). Quaternary glaciations of the southern Andes.. Quatern Sci Rev.

[11] Harrison S, Ehlers J, Gibbard PL (2004). The Pleistocene glaciations of Chile.. Developments in Quaternary Science: Elsevier.

[12] Potts R, Behrensmeyer AK, Behrensmeyer AK, Damuth JD, DiMichelle WA, Potts R, Sues HD (1992). Late Cenozoic terrestrial ecosystems.. Terrestrial ecosystems through time: evolutionary paleoecology of terrestrial plants and animals.

[13] Rabassa J, Rabassa J (2008). Late Cenozoic Glaciations in Patagonia and Tierra del Fuego.. The Late Cenozoic of Patagonia and Tierra del Fuego: Elsevier.

[14] Porter SC (1981). Pleistocene glaciation in the southern Lake District of Chile.. Quatern Res.

[15] Mercer JH (1983). Cenozoic Glaciation in the Southern Hemisphere.. Annu Rev Earth Planet Sci.

[16] Heusser CJ (1983). Quaternary pollen record from Laguna de Tagua Tagua, Chile.. Science.

[17] Villagran C (1988). Expansion of Magellanic moorland during the late Pleistocene; palynological evidence from northern Isla de Chiloé, Chile.. Quatern Res.

[18] Villagran C (1991). Historia de los bosques templados del sur de Chile durante el Tardiglacial y Postglacial.. Rev Chil Hist Nat.

[19] Villagran C, Hinojosa LF, Llorente-Bousquets J, Morrone JJ (2005). Esquema Biogeográfico de Chile. Regionalización Biogeográfica en Iberoamérica y Tópicos Afines.

[20] Holling JT, Schilling DH, Denton GH, Hughes TJ (1981). Late Wisconsin-Weichselian mountains glaciers and small ice caps.. The last great ice sheets.

[21] Villagran C, Hinojosa LF (1997). Historia de los bosques de Sudamérica, II: análisis fitogeográfico.. Rev Chil Hist Nat.

[22] Villagran C (1988). Late Quaternary vegetation of southern Isla Grande de Chiloé, Chile.. Quatern Res.

[23] Villagran C (1985). Análisis palinológico de los cambios vegetacionales durante el Tardiglacial y Postglacial en Chiloé, Chile.. Rev Chil Hist Nat.

[24] Villagrán C, Moreno P, Villa R, Armesto JJ, Villagrán C, Arroyo MK (1996). Antecedentes palinológicos acerca de la historia cuaternaria de los bosques chilenos.. Ecología de Los Bosques Nativos de Chile.

[25] Heusser CJ (1972). On the occurrence of *Lycopodium fuegianum* during Late-Pleistocene Interstades in the Province of Osorno, Chile.. Bull Torrey Bot Club.

[26] Heusser CJ (1982). Palynology of cushion bogs of the Cordillera Pelada, Province of Valdivia, Chile.. Quatern Res.

[27] Heusser CJ (1993). Late-glacial of southern South America.. Quatern Sci Rev.

[28] Armesto JJ, Arroyo MTK, Hinojosa LF, Veblen TT, Young KR, Veblen TT, Orme AR, Young KR (2007). The Mediterranean Environment of Central Chile.. The Physical Geography of South America.

[29] Mann G (1978). Los pequeños mamíferos de Chile.. Gayana Zool.

[30] Osgood WH (1943). The mammals of Chile.

[31] Toro J, Vega JD, Khan AS, Mills JN, Padula P (1998). An outbreak of hantavirus pulmonary syndrome, Chile, 1997.. Emerg Infect Dis.

[32] Lopez N, Padula P, Rossi C, Lazaro ME, Franze-Fernandez MT (1996). Genetic identification of a new hantavirus causing severe pulmonary syndrome in Argentina.. Virology.

[33] Padula PJ, Edelstein A, Miguel SDL, Lopez NM, Rossi CM (1998). Hantavirus Pulmonary Syndrome outbreak in Argentina: Molecular evidence for person-to-person transmission of Andes virus.. Virology.

[34] Medina RA, Torres-Pérez F, Galeno H, Navarrete M, Vial PA (2009). Ecology, genetic diversity, and phylogeographic structure of Andes virus in humans and rodents in Chile.. J Virol.

[35] Torres-Pérez F, Navarrete-Droguett J, Aldunate R, Yates TL, Mertz GJ (2004). Peridomestic small mammals associated with confirmed cases of human hantavirus disease on southcentral Chile.. Am J Trop Med Hyg.

[36] Dragoo JW, Lackey JA, Moore KE, Lessa EP, Cook JA (2006). Phylogeography of the deer mouse (*Peromyscus maniculatus*) provides a predictive framework for research on hantaviruses.. J Gen Virol.

[37] Rivera PC, Gonzales-Ittig RE, Rossi-Fraire HJ, Levis S, Gardenal CN (2007). Molecular identification and phylogenetic relationships among the species of the genus Oligoryzomys (Rodentia, Cricetidae) present in Argentina, putative reservoirs of hantaviruses.. Zool Scr.

[38] Torres-Pérez F, Palma RE, Hjelle B, Ferres M, Cook JA (2010). Andes virus infections in the rodent reservoir and in humans vary across contrasting landscapes in Chile.. Infect Genet Evol.

[39] Hjelle B, Torres-Pérez F (2010). Hantaviruses in the Americas and their role as emerging pathogens.. Viruses.

[40] Torres-Pérez F, Palma RE, Hjelle B, Holmes EC, Cook JA (2011). Spatial but not temporal co-divergence of a virus and its mammalian host.. Mol Ecol.

[41] Cosacov A, Sérsic AN, Sosa V, Johnson LA, Cocucci AA (2010). Multiple periglacial refugia in the Patagonian steppe and post-glacial colonization of the Andes: the phylogeography of *Calceolaria polyrhiza*.. J Biogeogr.

[42] Soltis DE, Gitzendanner MA, Strenge DD, Soltis PS (1997). Chloroplast DNA intraspecific phylogeography of plants from the Pacific Northwest of North America.. Plant Syst Evol.

[43] Petit RJ, Aguinagalde I, de Beaulieu JL, Bittkau C, Brewer S (2003). Glacial refugia: hotspots but not melting pots of genetic diversity.. Science.

[44] Vendramin GG, Anzidei M, Madaghiele A, Bucci G (1998). Distribution of genetic diversity in Pinus pinaster Ait. as revealed by chloroplast microsatellites.. Theor Appl Genet.

[45] Olsen JL, Stam WT, Coyer JA, Reusch TB, Billingham M (2004). North Atlantic phylogeography and large-scale population differentiation of the seagrass Zostera marina L.. Mol Ecol.

[46] Gallardo MH, Palma RE (1990). Systematics of *Oryzomys longicaudatus* (Rodentia: Muridae) in Chile.. J Mammal.

[47] Palma RE (1987). Sistemática evolutiva del genero *Oryzomys* Baird en Chile (Rodentia: Cricetidae) [M.Sc.].

[48] Murúa R, Gonzales LA, Meserve PL (1986). Population ecology of *Oryzomys longicaudatus philippii* (Rodentia, Cricetidae) in southern Chile.. J Anim Ecol.

[49] Guillot G, Santos F, Estoup A (2008). Analysing georeferenced population genetics data with Geneland: a new algorithm to deal with null alleles and a friendly graphical user interface.. Bioinformatics.

[50] Guillot G, Estoup A, Mortier F, Cosson JF (2005). A spatial statistical model for landscape genetics.. Genetics.

[51] Guillot G, Mortier F, Estoup A (2005). Geneland: a computer package for landscape genetics.. Mol Ecol Notes.

[52] Grant WAS, Bowen BW (1998). Shallow population histories in deep evolutionary lineages of marine fishes: insights from sardines and anchovies and lessons for conservation.. J Hered.

[53] Cope JM (2004). Population genetics and phylogeography of the blue rockfish (Sebastes mystinus) from Washington to California.. Can J Fish Aquat Sci.

[54] Glasser NF, Jansson KN, Harrison S, Kleman J (2008). The glacial geomorphology and Pleistocene history of South America between 38°S and 56°S.. Quatern Sci Rev.

[55] Armesto JJ, Villagrán C, Aravena JC, Pérez C, Smith-Ramírez C, Enright NJ, Hill RS (1995). Conifer forest of the chilean Coastal Range.. Ecology of the southern conifers.

[56] Premoli AC, Kitzberger T, Veblen TT (2000). Isozyme variation and recent biogeographical history of the long-lived conifer *Fitzroya cupressoides*.. J Biogeogr.

[57] Muellner AN, Tremetsberger K, Stuessy T, Baeza CM (2005). Pleistocene refugia and recolonization routes in the southern Andes: insights from *Hypochaeris palustris* (Asteraceae, Lactuceae).. Mol Ecol.

[58] Mathiasen P, Premoli AC (2010). Out in the cold: genetic variation of *Nothofagus pumilio* (Nothofagaceae) provides evidence for latitudinally distinct evolutionary histories in austral South America.. Mol Ecol.

[59] Smith MF, Kelt DA, Patton JL (2001). Testing models of diversification in mice in the *Abrothrix olivaceus/xanthorhinus* complex in Chile and Argentina.. Mol Ecol.

[60] Rodriguez-Serrano E, Cancino RA, Palma RE (2006). Molecular phylogeography of *Abrothrix olivaceus* (Rodentia: Sigmodontinae) in Chile.. J Mammal.

[61] Rodriguez-Serrano E, Hernandez CE, Palma RE (2008). A new record and an evaluation of the phylogenetic relationships of *Abrothrix olivaceus markhami* (Rodentia: Sigmodontinae).. Mammal Biol.

[62] Palma RE, Cancino RA, Rodríguez-Serrano E (2010). Molecular systematics of *Abrothrix longipilis* (Rodentia: Cricetidae: Sigmodontinae) in Chile.. J Mammal.

[63] Kim I, Phillips CJ, Monjeau JA, Birney EC, Noack K (1998). Habitat islands, genetic diversity, and gene flow in a Patagonian rodent.. Mol Ecol.

[64] Pastorino MJ, Gallo LA (2002). Quaternary evolutionary history of Austrocedrus chilensis, a cypress native to the Andean-Patagonian forest.. J Biogeogr.

[65] Ruzzante DE, Walde SJ, Cussac VE, Dalebout ML, Seibert J (2006). Phylogeography of the Percichthyidae (Pisces) in Patagonia: roles of orogeny, glaciation, and volcanism.. Mol Ecol.

[66] Zemlak TS, Habit EM, Walde SJ, Carrea C, Ruzzante DE (2010). Surviving historical Patagonian landscapes and climate: molecular insights from *Galaxias maculatus*.. BMC Evol Biol.

[67] Jakob SS, Martinez-Meyer E, Blattner FR (2009). Phylogeographic analyses and paleodistribution modeling indicate pleistocene in situ survival of *Hordeum* species (Poaceae) in southern Patagonia without genetic or spatial restriction.. Mol Biol Evol.

[68] Xu J, Perez-Losada M, Jara CG, Crandall KA (2009). Pleistocene glaciation leaves deep signature on the freshwater crab *Aegla alacalufi* in Chilean Patagonia.. Mol Ecol.

[69] Lessa EP, D'Elia G, Pardinas UF (2010). Genetic footprints of late Quaternary climate change in the diversity of Patagonian-Fueguian rodents.. Mol Ecol.

[70] Maddison WP, Knowles LL (2006). Inferring phylogeny despite incomplete lineage sorting.. Syst Biol.

[71] Hewitt GM (2004). The structure of biodiversity - insights from molecular phylogeography.. Front Zool.

[72] Hewitt GM (1999). Post-glacial re-colonization of European biota.. Biol J Linn Soc.

[73] Runck AM, Cook JA (2005). Postglacial expansion of the southern red-backed vole (Clethrionomys gapperi) in North America.. Mol Ecol.

[74] Conroy CJ, Cook JA (2000). Phylogeography of a post-glacial colonizer: *Microtus longicaudus* (Rodentia: Muridae).. Mol Ecol.

[75] Fedorov VB, Goropashnaya AV, Boeskorov GG, Cook JA (2008). Comparative phylogeography and demographic history of the wood lemming (*Myopus schisticolor*): implications for late Quaternary history of the taiga species in Eurasia.. Mol Ecol.

[76] Cook JA, Dawson NG, MacDonald SO (2006). Conservation of highly fragmented systems: The north temperate Alexander Archipelago.. Biol Conserv.

[77] Kotlik P, Deffontaine V, Mascheretti S, Zima J, Michaux JR (2006). A northern glacial refugium for bank voles (*Clethrionomys glareolus*).. Proc Natl Acad Sci USA.

[78] Patton JL, Smith MF (1992). mtDNA phylogeny of Andean mice: a test of diversification across ecological gradients.. Evolution.

[79] Lessa EP, Cook JA, Patton JL (2003). Genetic footprints of demographic expansion in North America, but not Amazonia, during the Late Quaternary.. Proc Natl Acad Sci USA.

[80] Gonzalez-Ittig RE, Rossi-Fraire HJ, Cantoni GE, Herrero ER, Benedetti R (2010). Population genetic structure of long-tailed pygmy rice rats (*Oligoryzomys longicaudatus*) from Argentina and Chile based on the mitochondrial control region.. Can J Zool.

[81] Palma RE, Rodríguez-Serrano E, Rivera-Milla E, Hernandez CE, Salazar-Bravo J (2010). Phylogenetic relationships of the pygmy rice rats of the genus *Oligoryzomys* Bangs, 1900 (Rodentia, Sigmodontinae).. Zool J Linn Soc.

[82] Carleton MD, Musser GG (1989). Systematic studies in oryzomyine rodents (Muridae, Sigmodontinae): a synopsis of *Microryzomys*.. Bull Am Mus Nat Hist.

[83] Redford KH, Eisenberg JF (1992). Mammals of the Neotropics: the southern cone, Chile, Argentina, Uruguay, Paraguay.

[84] Kingman JF (1982). The coalescent.. Stochast Proces Appl.

[85] Posada D, Crandall KA (2001). Intraspecific gene genealogies: trees grafting into networks.. Trends Ecol Evol.

[86] Belmar-Lucero S, Godoy P, Ferres M, Vial P, Palma RE (2009). Range expansion of *Oligoryzomys longicaudatus* (Rodentia, Sigmodontinae) in Patagonian Chile, and first record of Hantavirus in the region.. Rev Chil Hist Nat.

[87] Palma RE, Torres-Pérez F, Boric-Bargetto D, Kelt D, Lessa EP, Salazar-Bravo J, Patton JL (2007). The ecology and evolutionary history of *Oligoryzomys longicaudatus* in southern South American.. The Quintessential Naturalist: Honoring the Life and Legacy of Oliver P Pearson.

[88] Gannon WL, Sikes RS (2007). Guidelines of the American Society of Mammalogists for the use of wild mammals in research.. J Mammal.

[89] Mills JN, Yates TL, Childs JE, Parmenter RR, Ksiazek TG (1995). Guidelines for working with rodents potentially infected with hantavirus.. J Mammal.

[90] Higgins DG, Thompson JD, Gibson TJ (1996). Using CLUSTAL for multiple sequence alignments.. Methods Enzymol.

[91] Librado P, Rozas J (2009). DnaSP v5: a software for comprehensive analysis of DNA polymorphism data.. Bioinformatics.

[92] Nei M (1987). Molecular Evolutionary Genetics.

[93] Ihaka R, Gentleman R (1996). R: A Language for Data Analysis and Graphics.. J Comput Graph Stat.

[94] Bryant D (2004). Neighbor-Net: An Agglomerative Method for the Construction of Phylogenetic Networks.. Mol Biol Evol.

[95] Dress AW, Huson DH (2004). Constructing splits graphs.. IEEE/ACM Trans Comput Biol Bioinform.

[96] Huson DH (2006). Application of Phylogenetic Networks in Evolutionary Studies.. Mol Biol Evol.

[97] Hein J, Scierup MH, Wiuf C (2005). Gene genealogies, variation and evolution.

[98] Drummond AJ, Rambaut A, Shapiro B, Pybus OG (2005). Bayesian coalescent inference of past population dynamics from molecular sequences.. Mol Biol Evol.

[99] Drummond AJ, Rambaut A (2007). BEAST: Bayesian evolutionary analysis by sampling trees.. BMC Evol Biol.

[100] Weinstock J, Willerslev E, Sher A, Tong W, Ho SYW (2005). Evolution, Systematics, and Phylogeography of Pleistocene Horses in the New World: A Molecular Perspective.. PLoS Biol.

[101] Posada D (2008). jModelTest: Phylogenetic Model Averaging.. Mol Biol Evol.

[102] Gelman A, Carlin JB, Stern HS, Rubin DB (2003). Bayesian data analysis.

[103] Suchard MA, Weiss RE, Sinsheimer JS (2001). Bayesian selection of continuous-time Markov chain evolutionary models.. Mol Biol Evol.

[104] Rambaut A, Drummond AJ (2009). http://beast.bio.ed.ac.uk/Tracer.

[105] Rajabi-Maham H, Orth A, Bonhomme F (2008). Phylogeography and postglacial expansion of Mus musculus domesticus inferred from mitochondrial DNA coalescent, from Iran to Europe.. Mol Ecol.

[106] Trucchi E, Sbordoni V (2009). Unveiling an ancient biological invasion: molecular analysis of an old European alien, the crested porcupine (*Hystrix cristata*).. BMC Evol Biol.

[107] Kuhner MK (2006). LAMARC 2.0: maximum likelihood and Bayesian estimation of population parameters.. Bioinformatics.

[108] Wares JP, Cunningham CW (2001). Phylogeography and historical ecology of the north atlantic intertidal.. Evolution.

[109] Fu YX (1997). Statistical tests of neutrality of mutations against population growth, hitchhiking and background selection.. Genetics.

[110] Tajima F (1989). The effect of change in population size on DNA polymorphism.. Genetics.

[111] Ramos-Onsins SE, Rozas J (2002). Statistical properties of new neutrality tests against population growth.. Mol Biol Evol.

