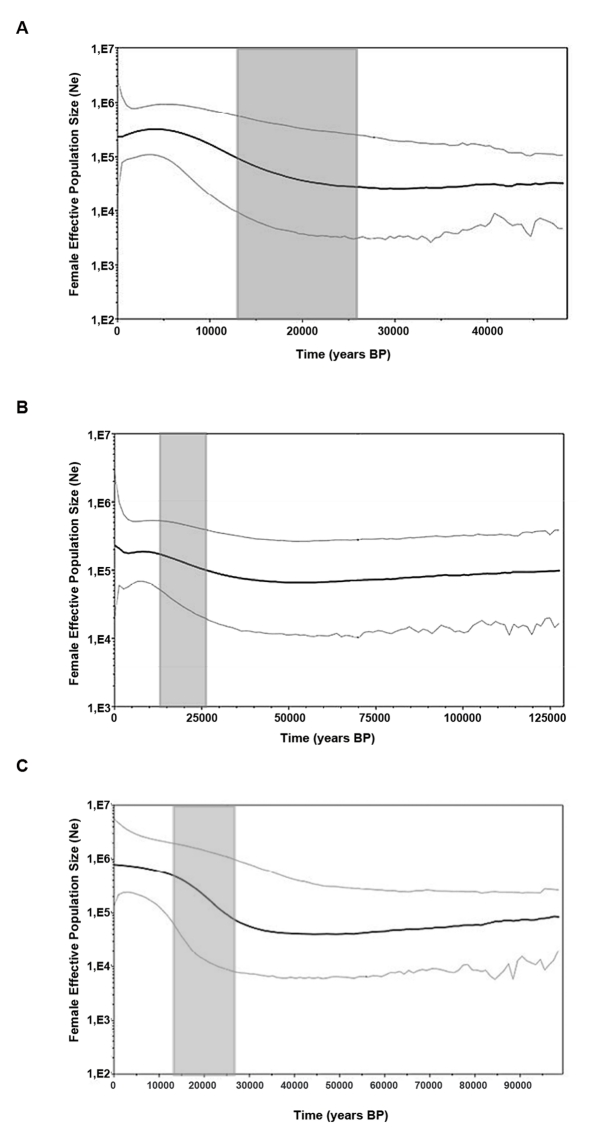# Correction: Glaciation Effects on the Phylogeographic Structure of *Oligoryzomys longicaudatus* (Rodentia: Sigmodontinae) in the Southern Andes

**DOI:** 10.1371/annotation/cacb082c-c9f2-4ea6-b1b5-a933615921a2

**Published:** 2012-03-20

**Authors:** R. Eduardo Palma, Dusan Boric-Bargetto, Fernando Torres-Pérez, Cristián E. Hernández, Terry L. Yates

There was an error in Figure 4. The correct Figure 4 can be viewed here: 

**Figure:**