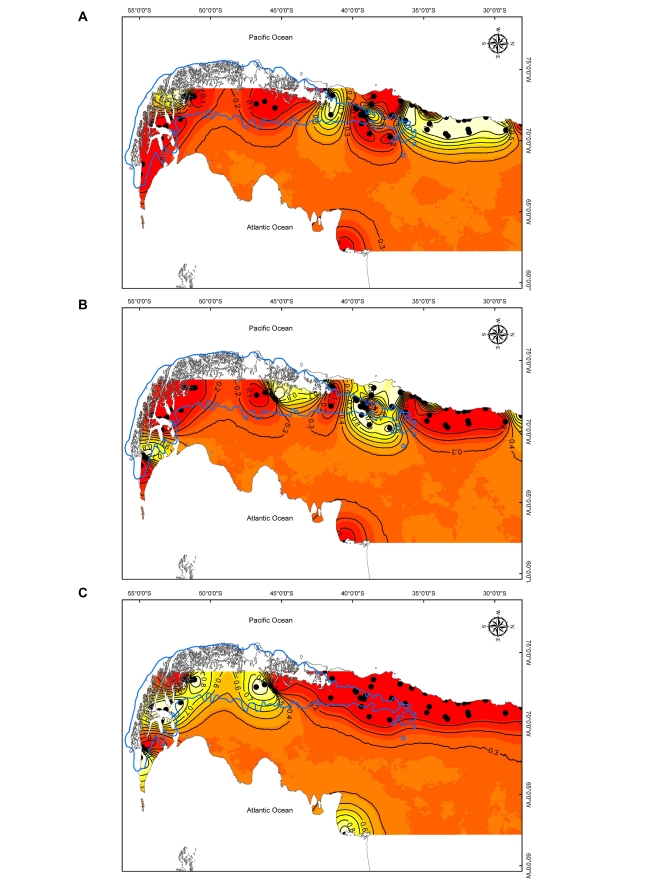# Correction: Glaciation Effects on the Phylogeographic Structure of *Oligoryzomys longicaudatus* (Rodentia: Sigmodontinae) in the Southern Andes

**DOI:** 10.1371/annotation/63d5ab3d-0a0b-4f9d-aca4-977cf3b4a0da

**Published:** 2012-03-20

**Authors:** R. Eduardo Palma, Dusan Boric-Bargetto, Fernando Torres-Pérez, Cristián E. Hernández, Terry L. Yates

There was an error in Figure 2. The correct Figure 2 can be viewed here: 

**Figure:**