# Supplementary material for: Glaciation Effects on the Phylogeographic Structure of Oligoryzomys longicaudatus (Rodentia: Sigmodontinae) in the Southern Andes
Source: PLoS One. 2012 Mar 1;7(3):e32206. doi: 10.1371/journal.pone.0032206 (PMC3291571; doi:10.1371/journal.pone.0032206)
Supplement: Table S1 — List of geographic localities analyzed in this study. Refer to numbers depicted in the map for localities (Fig. 1). N = sample size for that locality. (DOCX) [file pone.0032206.s001.docx]

**Table S1.-** **List of geographic localities analyzed in this study. Refer to numbers depicted in the map for localities** (Fig. 1). N = sample size for that locality.

| Locality | Locality # | Geographic Coordinates | N |
| --- | --- | --- | --- |
| CHILE  Mediterranean ecoregion (red numbers) |  |  |  |
| Atacama, Parque Nacional Llanos de Challe | 1 | 28˚ 05' 02" S, 71˚ 08´ 25" W | 2 |
| Coquimbo, Observatorio La Silla | 2 | 29˚ 13' 58" S, 70˚ 44´ 14" W | 4 |
| Coquimbo, Parque Nacional Fray Jorge | 3 | 30˚ 38' 58" S, 71˚ 41´ 14" W | 24 |
| Coquimbo, Minera Pelambres | 4 | 31˚ 49' 13" S, 70˚ 34´ 56" W | 4 |
| Coquimbo, Chillepín, Salamanca | 5 | 31˚ 53' 12." S, 70˚ 47´ 47" W | 1 |
| Valparaíso, Cerro Santa Inés | 6 | 32˚ 08' 50" S, 71˚ 27´ 35" W | 10 |
| Coquimbo, Los Vilos | 7 | 32˚ 09' 29" S, 71˚ 29´ 02" W | 12 |
| Valparaíso, Quebrada del Tigre | 8 | 32˚ 33' 36" S, 71˚ 26´ 19" W | 11 |
| Valparaíso, Quebrada de Córdova | 9 | 33˚ 26' 18" S, 71˚ 39´ 14" W | 6 |
| Región Metropolitana, Santiago, Yerba Loca | 10 | 33˚ 18' 40" S, 70˚ 19´ 31" W | 3 |
| Región Metropolitana, Santiago, San Carlos de Apoquindo | 11 | 33˚ 28' 08" S, 70˚ 29´ 07" W | 19 |
| O’Higgins, San Fernando, Las Peñas | 12 | 34˚ 45' 59" S, 70˚ 46´ 35” W | 1 |
| Maule, Curicó, El Trapiche | 13 | 34˚ 35' 24" S, 71˚ 11´ 24” W | 1 |
| Maule, Curicó, Duao | 14 | 34˚ 52' 55" S, 72˚ 09´ 15” W | 2 |
| Maule, Reserva Nacional Los Ruiles | 15 | 35˚ 50’ 02” S, 72˚ 30´ 34”W | 5 |
| Maule, Bullileo | 16 | 36˚ 17’ 20” S, 71˚ 24´ 47” W | 3 |
| Bío-Bío, Tomé | 17 | 36˚ 38' 29" S, 72˚ 47´ 46” W | 2 |
| Bío-Bío, Tucapel  Temperate Forests ecoregion (green numbers) | 18 | 37˚ 14’ 28” S, 71˚ 47´ 38” W | 2 |
| Araucanía, Carahue | 19 | 38˚ 30’ 55” S, 73˚ 06´ 59” W | 4 |
| Araucanía, Temuco, Fundo Chivilcán | 20 | 38˚ 41' 06" S, 72˚ 36´ 27” W | 1 |
| Araucanía, Lago Colico | 21 | 39˚ 03’ 51” S, 71˚ 58´ 24” W | 1 |
| Araucanía, Parque Nacional Huerquehue | 22 | 39˚ 10’ 10” S, 71˚ 43´ 36” W | 1 |
| Araucanía, Parque Nacional Villarrica | 23 | 39˚ 27’ 28” S, 71˚ 49´ 33” W | 29 |
| Araucanía, Quetrupillán | 24 | 39˚ 25’ 38” S, 71˚ 47´ 16” W | 7 |
| Los Ríos, Panguipulli | 25 | 39˚ 44’ 12” S, 72˚ 13´ 39” W | 13 |
| Los Ríos, Riñihue | 26 | 39˚ 48’ 15” S, 72˚ 19´ 15” W | 6 |
| Los Lagos, Puerto Montt, Las Quemas | 27 | 41˚ 24’ 57” S, 73˚ 11´ 27” W | 3 |
| Los Lagos, Llanquihue, Paso El León | 28 | 41˚ 31' 55" S, 71˚ 49´ 50” W | 1 |
| Los Lagos, Chiloé, Senda Darwin  Patagonian ecoregion (blue numbers) | 29 | 41˚ 52’ 57” S, 73˚ 40´ 08” W | 4 |
| Aysén, Reserva Nacional Río Simpson | 30 | 45˚ 27’ 42” S, 72˚ 19´ 21” W | 6 |
| Aysén, Río Ibañez, Lago Cofré | 31 | 46˚ 11' 22" S, 72˚ 46´ 32” W | 1 |
| Aysén, Chile Chico, Puerto Guadal | 32 | 46˚ 46’ 48” S, 72˚ 36´ 09” W | 3 |
| Magallanes, Parque Nacional Torres del Paine | 33 | 51˚ 07’ 24” S, 73˚ 07´ 47” W | 5 |
| Magallanes, Reserva Nacional Alacalufe | 34 | 51˚ 52’ 06” S, 73˚ 08´ 02” W | 2 |
| Magallanes, Río Penitente | 35 | 52˚ 06’ 45” S, 71˚ 32´ 10” W | 1 |
| Magallanes, Isla Riesco | 36 | 52˚51’48” S, 71˚32’45” W | 6 |
| Magallanes, Reserva Nacional Magallanes | 37 | 53˚07’59” S, 71˚01’30” W | 3 |
| Magallanes, Tierra del Fuego, Porvenir | 38 | 53˚ 27' 49" S, 70˚ 10´ 52" W | 1 |
| Magallanes, Puerto de Hambre | 39 | 53˚36’09” S, 70˚56’26” W | 3 |
| Magallanes, Fuerte Bulnes | 40 | 53˚ 37' 42" S, 70˚ 55´ 19” W | 2 |
| Magallanes, Parque Omora | 41 | 54˚ 57’ S, 67˚ 39´ W | 1 |
| Magallanes, Bahía Inútil | 42 | 54˚ 59’ S, 68˚ 13´ W | 1 |
|  |  |  |  |
| ARGENTINA |  |  |  |
| Neuquén, Chos Malal | 43 | 37˚ 23' 15" S, 70˚ 16´ 40" W | 1 |
| Neuquén, Cañadón Santo Domingo | 44 | 38˚ 50' S, 70˚ 30´ W | 1 |
| Neuquén, Las Breñas | 45 | 39˚ 23' S, 71˚ 12´ W | 1 |
| Buenos Aires, Bahía San Blas | 46 | 40˚ 33' S, 62˚ 13 W | 2 |
| Ushuaia, Tierra del Fuego (10 km W of Ushuaia) | 47 | 54˚ 47' 03” S, 68˚ 23´ 01” W | 1 |
