# Supplementary material for: Glaciation Effects on the Phylogeographic Structure of Oligoryzomys longicaudatus (Rodentia: Sigmodontinae) in the Southern Andes
Source: PLoS One. 2012 Mar 1;7(3):e32206. doi: 10.1371/journal.pone.0032206 (PMC3291571; doi:10.1371/journal.pone.0032206)
Supplement: Table S2 — List of haplotypes recovered through the mitochondrial control region (hypervariable I domain) for Oligoryzomys longicaudatus . In parenthesis the number of haplotypes. The localities (see Table 1 for geographic details) are associated to specimens trapped in that site. The NK is a collection number used for the Division of Biological Materials of the Museum of Southwestern Biology, University of New Mexico, USA, and the Departamento de Ecología, Pontificia Universidad Católica de Chile, Chile. Other acronyms used are UP (field catalog of Dr. Ulyses Pardiñas), JCT (Juan Carlos Torres-Mura). (DOC) [file pone.0032206.s002.doc]

**Table S2**. Complete list of haplotypes recovered through the mitochondrial control region (hypervariable I domain) for *Oligoryzomys longicaudatus*. In parenthesis the number of haplotypes of that particular haplotype. The localities are associated to specimens trapped in that site (see Table 1 for geographic details). The NK is a collection number used for the Division of Biological Materials of the Museum of Southwestern Biology, University of New Mexico, USA, and the Departamento de Ecología, Pontificia Universidad Católica de Chile, Chile. Other acronyms used are UP (field catalog of Dr. Ulyses Pardiñas); SSUC (Colección de Flora y Fauna Prof. Patricio Sánchez, Pontificia Universidad Católica de Chile; JCT (field catalog of Juan Carlos Torres-Mura); CZIP (Colección de Zoología, Instituto de la Patagonia, Magallanes, Chile).

Haplotype 1 (10): Parque Nacional Fray Jorge 164, NK 96907, NK 96912, NK 109220; Cerro Santa Inés NK 120736; Quebrada del Tigre NK 96772; Minera Pelambres NK 96603; Los Vilos NK 108858, NK 108865, NK 120730.

Haplotype 2 (1): Parque Nacional Fray Jorge NK 95728.

Haplotype 3 (3): Parque Nacional Fray Jorge NK 95735, NK 96896, NK 96921.

Haplotype 4 (1): Parque Nacional Fray Jorge NK 95736.

Haplotype 5 (6): Parque Nacional Fray Jorge NK 96867, NK 96876, NK 96898, NK 109257, NK 96865, NK 96887.

Haplotype 6 (7): Parque Nacional Fray Jorge NK 96890, NK 96892, NK 96914, NK 96919, NK 96920, NK109264, Cerro Santa Inés105345]

Haplotype 7 (5): Parque Nacional Fray Jorge NK 96916, NK 96917; Quebrada de Córdova NK 105408; Minera Pelambres NK 96590, NK 96601.

Haplotype 8 (1): Cerro Santa Inés NK 105340

Haplotype 9 (2): Cerro Santa Inés NK 105343; San Carlos de Apoquindo NK 108877

Haplotype 10 (1): Cerro Santa Inés NK 105346

Haplotype 11 (1): Cerro Santa Inés NK 105347

Haplotype 12 (1): Cerro Santa Inés NK 120734

Haplotype 13 (1): Cerro Santa Inés NK 120735

Haplotype 14 (8): Cerro Santa Inés: NK 120739, NK 120740; San Carlos de Apoquindo NK 95294, NK 96813, NK 96833, NK 104647, NK 105004, NK 105573

Haplotype 15 (1): Quebrada del Tigre NK 96743

Haplotype 16 (8): Quebrada del Tigre NK 96745, NK 96751, NK 96756, NK 96757, NK 96762; Los Vilos NK 108857, NK 108862; Reserva Nacional Alacalufe NK 108796

Haplotype 17 (8): Quebrada del Tigre NK 96758; San Carlos de Apoquindo NK 95693, NK 104605; Reserva Nacional Los Ruiles NK 108830, NK 108840, NK 108841; Parque Nacional Villarrica NK 105137; Tomé NK 120077

Haplotype 18 (1): Quebrada del Tigre NK 96759

Haplotype 19 (3): Quebrada del Tigre NK 96764, NK 96773; Parque Nacional Torres del Paine NK 105659

Haplotype 20 (1): Quebrada de Córdova NK 105406

Haplotype 21 (2): Quebrada de Córdova NK 108879; Panguipulli NK 104535

Haplotype 22 (1): Quebrada de Córdova NK 108880

Haplotype 23 (2): Quebrada de Córdova NK 108882; Bullileo NK 105879

Haplotype 24 (1): Quebrada de Córdova NK 108883

Haplotype 25 (7): Llanos de Challe NK 105362; Quetrupillán NK 95367; Parque Nacional Villarrica NK 104795, NK 105014, NK 105144; Riñihue NK 104595; Parque Nacional Huerquehue 105362

Haplotype 26 (1): Llanos de Challe NK 105380

Haplotype 27 (4): Observatorio La Silla NK 96840, NK 96858, NK 96859, NK 96860

Haplotype 28 (1): Minera Pelambres NK 96594

Haplotype 29 (1): Los Vilos NK 108856

Haplotype 30 (2): Los Vilos NK 108860, NK 108864]

Haplotype 31 (1): Los Vilos NK 108869

Haplotype 32 (1): Los Vilos NK 120721

Haplotype 33 (1): Los Vilos NK 120728

Haplotype 34 (2): San Carlos de Apoquindo NK 95290, NK 104619

Haplotype 35 (4): San Carlos de Apoquindo NK 95299; Yerba Loca NK 108785, NK 108786, NK 108787

Haplotype 36 (1): San Carlos de Apoquindo NK 96838

Haplotype 37 (1): San Carlos de Apoquindo NK 104623

Haplotype 38 (1): San Carlos de Apoquindo NK 104644

Haplotype 39 (1): San Carlos de Apoquindo NK 104648

Haplotype 40 (1): San Carlos de Apoquindo NK 105566

Haplotype 41 (1): Bullileo NK 105880

Haplotype 42 (1): Bullileo NK 105885

Haplotype 43 (1): Carahue NK 95603

Haplotype 44 (1): Carahue NK 95612

Haplotype 45 (1): Carahue NK 95614

Haplotype 46 (1): Quetrupillán NK 95366

Haplotype 47 (3): Quetrupillán NK 95379, NK 95380; Panguipulli NK 96973

Haplotype 48 (8): Quetrupillán NK 95388; Parque Nacional Villarrica NK 104754, NK 104761, NK 104766, NK 105030, NK 95083; Panguipulli NK 105578; Temuco Fundo Chivilcán NK 96703

Haplotype 49 (1): Quetrupillán NK 95395

Haplotype 50 (2): Quetrupillán 95399; Parque Nacional Villarrica NK 96401

Haplotype 51 (8): Parque Nacional Villarrica NK 95048, NK 95427, NK 96403, NK 104759, NK 106497, NK 108827; Panguipulli NK 105584; Riñihue NK 104592

Haplotype 52 (3): Parque Nacional Villarrica NK 95060, NK 95097, NK 95421

Haplotype 53 (5): Parque Nacional Villarrica NK 96420, NK 104764, NK 105147; Puerto Montt Las Quemas NK 105157, NK 105165

Haplotype 54 (1): Parque Nacional Villarrica NK 104763

Haplotype 55 (3): Parque Nacional Villarrica NK 104765; Riñihue NK 104593; Carahue NK 95613

Haplotype 56 (2): Parque Nacional Villarrica 104775; Las Breñas LBO 12

Haplotype 57 (1): Parque Nacional Villarrica NK 104779

Haplotype 58 (2): Parque Nacional Villarrica NK 105015; Chiloé Senda Darwin NK 95646

Haplotype 59 (3): Parque Nacional Villarrica NK 105044; Panguipulli NK 105839; Tomé NK 120076

Haplotype 60 (2): Parque Nacional Villarrica NK 105148; Cañadón Santo Domingo UP 435

Haplotype 61 (1): Panguipulli NK 96972

Haplotype 62 (2): Panguipulli NK 105577, NK 105838

Haplotype 63 (1): Panguipulli NK 105837

Haplotype 64 (1): Panguipulli NK 105585

Haplotype 65 (1): Riñihue NK 104559

Haplotype 66 (1): Riñihue NK 104594

Haplotype 67 (1): Riñihue NK 104565

Haplotype 68 (1): Chiloé Senda Darwin NK 95638

Haplotype 69 (1): Chiloé Senda Darwin NK 95639

Haplotype 70 (1): Chiloé Senda Darwin NK 95640

Haplotype 71 (2): Puerto Montt Las Quemas NK 105168; Panguipulli NK 104515

Haplotype 72 (2): Reserva Nacional Río Simpson NK 96548, NK 96565

Haplotype 73 (3): Reserva Nacional Río Simpson NK 96560, NK 96555, NK 96566

Haplotype 74 (1): Reserva Nacional Río Simpson NK 96562

Haplotype 75 (1): Río Penitente, CZIP 1025

Haplotype 76 (1): Parque Nacional Torres del Paine NK 105649

Haplotype 77 (1): Parque Nacional Torres del Paine NK 105650

Haplotype 78 (1): Reserva Nacional Alacalufe NK 108795

Haplotype 79 (2): Chos Malal UP 449; Tucapel NK 105959

Haplotype 80 (2): Bahía San Blas UP 374, UP 377

Haplotype 81 (1): Parque Omora, JCT 1950

Haplotype 82 (1): Bahía Inútil, JCT 1960

Haplotype 83 (1): Parque Nacional Torres del Paine NK 105653

Haplotype 84 (1): Parque Nacional Torres del Paine NK 105667

Haplotype 85 (1): Reserva Nacional Magallanes NK 129230

Haplotype 86 (1): Reserva Nacional Magallanes NK 129229

Haplotype 87 (1): Reserva Nacional Magallanes NK 129245

Haplotype 88 (1): Fuerte Bulnes NK 129291

Haplotype 89 (1): Fuerte Bulnes NK 129342

Haplotype 90 (2): Puerto de Hambre NK 129284, NK 129292

Haplotype 91 (1): Puerto de Hambre NK129290

Haplotype 92 (1): Isla Riesco NK 142506

Haplotype 93 (1): Isla Riesco NK 142507

Haplotype 94 (1): Isla Riesco NK 142513

Haplotype 95 (1): Isla Riesco NK 142514

Haplotype 96 (1): Isla Riesco NK 142529

Haplotype 97 (1): Isla Riesco NK 142530

Haplotype 98 (2): Tierra del Fuego Ushuaia SSUC 00406, Porvenir NK160209

Haplotype 99 (1): Chillepín Salamanca NK 95621

Haplotype 100 (1): San Carlos de Apoquindo NK 96325

Haplotype 101 (1): Panguipulli NK 96979

Haplotype 102 (1): Panguipulli NK 104544

Haplotype 103 (1): San Carlos de Apoquindo NK 105110

Haplotype 104 (1): Lago Cofré 105306

Haplotype105 (1): San Fernando Las Peñas NK 105929

Haplotype 106 (1): Tucapel NK 105979

Haplotype107 (1): Duao NK 108501

Haplotype 108 (1): Duao NK 108505

Haplotype 109 (1): Reserva Nacional Los Ruiles NK 108843

Haplotype 110 (1): Reserva Nacional Los Ruiles NK 108853

Haplotype 111 (1): Los Vilos NK 108859

Haplotype 112 (1): Parque Nacional Fray Jorge NK 109249

Haplotype 113 (1): Curicó El Trapiche NK 120092

Haplotype 114 (1): Puerto Guadal NK 120378

Haplotype 115 (2): Puerto Guadal NK 120386, NK 120387]

Haplotype 116 (1): Paso El León NK 129454

Haplotype 117 (1): Lago Colico NK 160300
